# Objective Assessment of Physical Activity in Breast Cancer Survivors: Associations With Adiposity and Metabolic Parameters of Glucose and Insulin—Insights From NHANES

**DOI:** 10.1002/cnr2.70339

**Published:** 2025-09-12

**Authors:** Jose J. Cao‐Alvira, Emilia F. Luciano, Moira Sauane, Columba de la Parra

**Affiliations:** ^1^ Department of Finance, Information Systems and Economics, Herbert H. Lehman College City University of New York New York New York USA; ^2^ Department of Economics, Barnard College Columbia University New York New York USA; ^3^ Department of Chemistry, Herbert H. Lehman College City University of New York New York New York USA; ^4^ Department of Biological Sciences, Herbert H. Lehman College City University of New York New York New York USA; ^5^ City University of New York New York New York USA; ^6^ Ph.D. Program in Chemistry City University of New York New York New York USA

**Keywords:** body mass index (BMI), breast cancer, cancer survivors, glucose and insulin metabolism parameters, machine learning, NHANES, physical activity, sagittal abdominal diameter (SAD), waist circumference (WC)

## Abstract

**Background:**

Physical activity (PA) is a modifiable lifestyle factor strongly linked to adiposity and metabolic health. Breast cancer survivors are at a higher risk of obesity‐related metabolic complications, negatively impacting prognosis and quality of life. Therefore, updated objective assessments of PA and its effects on metabolic health are critical to improving survivorship care.

**Aims:**

To analyze the most recent nationally representative sample of the US population where physical activity was objectively measured to identify associations between PA, adiposity measures, and metabolic health parameters among cancer survivors, specifically breast cancer survivors.

**Methods and Results:**

NHANES data from 2011 to 2014 is used to examine associations between PA, adiposity measures, and metabolic parameters in women aged 20 and older. PA is objectively measured using accelerometer data. Adiposity is assessed via sagittal abdominal diameter (SAD), waist circumference (WC), and body mass index (BMI), and the metabolic parameters are glucose and insulin. The analysis involved regressing the natural logarithms of SAD, WC, BMI, glucose, and insulin on moderate‐to‐vigorous and sedentary PA levels. The sample consisted of 4068 participants, including 397 with cancer diagnoses and 118 with breast cancer. Additionally, factors such as ethnicity, education, income, age, energy intake, diabetes, and smoking habits were considered. The associations between adiposity measures and excess daily sedentary PA are stronger for breast cancer survivors compared to all women and cancer survivors, with statistically significant results for SAD. SAD directly assesses visceral fat and is more closely linked to metabolic and cardiovascular risks. While moderate‐to‐vigorous PA is associated with decreased adiposity for all women, the decrease is less pronounced among cancer survivors. Daily moderate‐to‐vigorous PA has a significant and negative correlation with glucose and insulin levels, whereas sedentary PA has a positive correlation. The associations of PA with metabolic parameters in breast cancer survivors are similar to those in all women and cancer survivors.

**Conclusion:**

This study provides an updated objective assessment of PA in breast cancer survivors using the most recent NHANES survey cycles, where this data was collected. The results confirm rising obesity rates among breast cancer survivors, highlighting the need for ongoing monitoring of adiposity. The results suggest that engaging in daily moderate‐to‐vigorous PA significantly lowers glucose and insulin levels, which are crucial for metabolic health. Given its modifiable nature, PA can be a valuable intervention for breast cancer patients, emphasizing the importance of its assessment. Implementing lifestyle changes that prioritize PA and targeted interventions to manage SAD is essential, as reducing sedentary behavior can lead to improved health outcomes with significant public health implications.

## 1 Introduction

Cancer continues being a global public health challenge and, with over 2.3 million new annual cases, breast cancer is the most diagnosed cancer among women worldwide. In the United States, the number of breast cancer survivors exceeds 4 million. Breast cancer is the second leading cause of cancer‐related deaths in women, with 42 250 yearly deaths in the Unites States, following lung cancer [1, 2]. Mortality rates have consistently decreased since the 1980s, reflecting improvements in early detection and treatment strategies [3].

Weight gain and metabolic syndrome are common among cancer survivors^1^, affecting their physical well‐being and quality of life [4, 5, 6]. Excess weight and adipose tissue, particularly visceral fat distribution, are linked to increased cancer recurrence risk. Overweight and obese cancer survivors often encounter additional obstacles that could compromise treatment efficacy [5, 7, 8, 9]. Recent population‐based studies in the United States have highlighted a concerning rise in obesity rates among cancer survivors, notably impacting those diagnosed with breast cancer [10, 11]. The prevalence of diabetes among this population is concerning [12]. Survivors with metabolic syndrome,^2^ characterized by abdominal obesity, hyperglycemia, dyslipidemia, and hypertension (and often accompanied by insulin resistance), experience a higher incidence of breast cancer recurrence. Metabolic syndrome is associated with the recurrence of breast cancer in survivors through mechanisms such as obesity, physical inactivity, hyperinsulinemia, and insulin resistance [13, 14]. Moreover, following chemotherapy, metabolic syndrome is further aggravated in breast cancer patients, increasing their susceptibility to mortality from cardiovascular diseases and metabolic disorders, including diabetes [14, 15]. Tamoxifen, a selective estrogen receptor modulator commonly prescribed for hormone receptor‐positive breast cancer, has been associated with impaired glucose metabolism and weight gain. These metabolic disturbances are particularly evident in women who are overweight or obese and may be further intensified during the menopausal transition [14, 16, 17]. Studies have shown that tamoxifen use can reduce insulin sensitivity and may even promote apoptosis of pancreatic β‐cells, contributing to an increased risk of type 2 diabetes mellitus and increased visceral adipose tissue, as reflected by elevated sagittal abdominal diameter (SAD) [18, 19]. As breast cancer survivors age and undergo natural hormonal transitions, these treatment‐related effects may further compromise weight regulation and metabolic health [20, 21]. These findings emphasize the importance of closely monitoring metabolic parameters and promoting healthy lifestyle behaviors as essential components of survivorship care.

Physical activity (PA) emerges as a critical determinant of health outcomes in cancer survivors, particularly in managing recurrence risks and concurrent health conditions. Established risk factors—such as excess body weight and physical inactivity—are influential on the cancer prognosis [5, 22]. Recent works in the literature emphasize the association between prolonged sedentary behavior and impaired glucose metabolism, potentially intensifying the risks of metabolic syndrome and recurrent breast cancer [6, 23]. Extended periods of sedentary behavior correlate with higher mortality rates, type II diabetes, cardiovascular diseases, and cancer recurrence among survivors [24]. Evidence also suggests that sedentary behaviors may influence breast cancer progression through mechanisms involving insulin resistance, although the exact pathways are still under investigation [25, 26, 27].

There is increasing evidence indicating that incorporating regular light‐intensity PA into daily routines can significantly improve health and reduce risk factors [28, 29, 30]. PA is crucial for breast cancer survivorship, impacting both prognosis and quality of life. Light, moderate‐to‐vigorous, and total PA are robustly associated with reduced mortality risk post‐cancer diagnosis in a 10‐year follow‐up analysis [31]. Population‐based studies have also underscored PA's role in reducing adverse health outcomes among breast cancer survivors [32]. For instance, when compared to control groups in randomized controlled trials, sedentary and overweight breast cancer survivors experienced significant improvements in metabolic syndrome, obesity, and other various biomarkers after supervised programs that included moderate‐to‐vigorous aerobic exercise [33].

Since physical activity is a modifiable lifestyle risk factor, it could serve as a viable intervention for breast cancer patients. Monitoring and understanding the PA levels of this population is critical. Breast cancer survivorship research has historically relied on self‐reported measures of PA, providing valuable data [34]. Nevertheless, relying solely on patient self‐reporting for PA assessment has shown susceptibility to recall errors and over‐reporting [6]. This limitation has effectively been addressed through the use of accelerometers, which are compact devices that objectively measure PA in population‐based studies by recording acceleration data. Accelerometers have revolutionized the objective assessment of PA by providing accurate data that can be analyzed to offer detailed insights into daily activity patterns.

Using data from the National Health and Nutrition Examination Survey (NHANES), we examine the associations of PA with adiposity measures and glucose and insulin metabolism parameters among women and cancer survivors, focusing particularly on breast cancer survivors. NHANES, administered by the Centers for Disease Control and Prevention's National Center for Health Statistics (NCHS), employs a rigorous multistage sampling design to ensure a nationally representative sample of the US population. The significance of PA for breast cancer survivors has been well established and remains a dynamic area of ongoing research.

While drawing valuable insights from existing studies, this research is the first to utilize the most recent NHANES implementation cycles (2011–12 and 2013–14) where objectively measured physical activity was collected by the national survey. A key advancement in these cycles over previous implementations is the shift from hip‐worn uniaxial accelerometers to wrist‐worn triaxial ActiGraph GT3X+ devices. This transition allows for more precise and continuous monitoring of movement across multiple axes, capturing activities previously underreported, such as upper‐body and aquatic activities. These methodological improvements address limitations of both self‐reported measures and earlier device‐based approaches, enhancing the accuracy and scope of PA assessments. By using this enhanced data, our study presents a unique opportunity to explore the relationship between objectively measured PA, adiposity, and metabolic health in breast cancer survivors.

This manuscript analyzes accelerometer data to measure the impact of PA on adiposity measures, specifically SAD, waist circumference (WC) and body mass index (BMI), as well as parameters related to glucose and insulin metabolism. The dataset, covering from 2011 to 2014, provides a comprehensive basis for analyzing detailed PA patterns across the general population, cancer survivors, and breast cancer survivors. Our analysis shows that, when compared to all women and all cancer survivors that participated in the study, the associations between adiposity measures and excess daily sedentary PA are amplified for breast cancer survivors. Moderate‐to‐vigorous PA is associated with decreases in adiposity measures for all women in the study, but to a lesser degree among breast cancer survivors. Engaging in daily moderate‐to‐vigorous PA is associated with significant decreases in the levels of two critical metabolic parameters—glucose and insulin. The opposite is true when engaging in daily sedentary PA. Distinct from what was observed between PA and adiposity, the associations of PA and the metabolic parameters for breast cancer survivors are not distinct from those observed among all women and all cancer survivors.

Our findings emphasize the clinical value of incorporating objective physical activity monitoring into care for breast cancer survivors. By focusing on modifiable lifestyle risk factors, particularly sedentary behaviors, we can enhance our understanding of how behavioral patterns influence metabolic health in this population. These findings support the development of targeted interventions and influence clinical practices aimed at improving long‐term health outcomes for breast cancer survivors.

## 2 Data and Methods

The CDC's NHANES conducts a complex multistage probability sampling and collects data on the health characteristics of the noninstitutionalized US population in the 50 states and DC [35]. Comprehensive individual‐level health data are collected during two‐year cycles through structured questionnaires, physical examinations, and laboratory testing protocols. All participants provided written informed consent, and the study protocols were approved by the CDC and the National Center for Health Statistics Ethics Review Board.^3^ The 2011–12 and 2013–14 are the most recent cycles of the NHANES survey when objectively measured physical activity was collected among its participants. This manuscript focuses on the demographic, anthropometric, glycemic, and behavioral data collected on the 20 years or older female participants of the survey during these two cycles. The sample includes 5833 participants before the removal of missing values. Following this, we describe the data collection and cleanup process.

### 2.1 Objectively Measured Physical Activity

Objectively measured data on PA was collected from each surveyed participant wearing a physical activity monitor continuously for 9 days. Participants started wearing the monitor immediately after their initial examination at the Mobile Examination Center (MEC) and removed it on the morning of the ninth day. Participants wore the device on their non‐dominant wrist using a custom‐fitted mesh wristband and were instructed to wear it consistently throughout the monitoring period. The ActiGraph model GT3X+ was selected for its triaxial measurement capabilities, extended battery life, and water resistance. Data were initially collected in ActiGraph GT3X+ format and presented in Monitor‐Independent Movement Summary (MIMS) units per minute. MIMS quantifies the continuous movements of the participants, where higher values represent higher activity.

PA data collected for each participant on the first and ninth day of the examination period is not considered in the analysis. Those days when a participant wore the physical activity monitor for less than 10 h were also eliminated from the sample. All PA observations originally flagged due to invalid measurements were eliminated from the study sample, as well as observations recording PA above the 99th percentile that were eliminated from the sample.

The survey contains an indicator variable for each minute of the day predicting if the participant is “wake,” “sleep,” “non‐wear,” or “unknown.” Provided that the focus of the analysis is on physical activity, the PA data were limited to those time intervals of the day when the participant was classified as “wake.” This resulted in an average of 14.9 h of daily PA monitoring. Quartiles of the PA data were computed, for example, {Q1, Q2, Q3, Q4}, where Q1 represents the quartile with the least PA and Q4 the highest. Each minute of PA per participant per day is classified within a quartile. The MIMS threshold delimiting Q1 and Q2 is 5.502, for Q2 and Q3 is 13.189, and for Q3 and Q4 is 23.509. Q4 and Q1 broadly represent, respectively, moderate‐to‐vigorous physical activity and sedentary activity.

### 2.2 Anthropometric Measurements

SAD, WC, and BMI measurements were obtained at the MEC by trained health technicians during the initial examination. Pregnant women and individuals weighing more than 600 pounds were excluded from the sample. SAD was measured using an abdominal caliper. Participants lay horizontally on an exam table with relaxed flexed hips. The examiner marked their iliac crests with a wax pencil and positioned the caliper accordingly. After aligning and confirming vertical placement, the examiner instructed participants to inhale gently, exhale slowly, and pause. The caliper's upper arm was then slid down to lightly touch the abdomen without compression, and the SAD value was recorded directly from the caliper's scale. WC was measured while standing, with the measurement tape positioned between the bottom of the ribs and the top of the iliac crest, parallel to the floor, at the conclusion of a normal exhalation. The tape was applied closely against the skin without exerting pressure during the measurement. BMI was calculated by dividing weight in kilograms by height in meters squared, rounded to one decimal place (kg/m^2^) [36].

### 2.3 Cancer Survivorship and Additional Covariates

Data availability and cleanup reduced the sample size to a total of 4068 participants. Each participant reported if they have ever been diagnosed with cancer, and which type; of which, 397 participants were diagnosed with cancer, of which 118 were breast cancer. To control the analysis for potential confounding variables, self‐reported data on ethnicity, education, income, age, energy intake, diabetes, and smoking habits were collected for each participant.

Table 1 contains descriptive statistics of the anthropometric, behavioral, and demographic characteristics of the individuals surveyed in NHANES 2011–12 and 2013–2014 selected for the study. Mean values are presented for all participants (*n* = 4068), for all participants that are not cancer survivors (*n* = 3671), for cancer survivors (*n* = 397) and for breast cancer survivors (*n* = 118). PA measures are presented as the average minutes per day spent on each quartile. The three adiposity measures are greater for breast cancer survivors, as well as the daily minutes spent in low daily sedentary activities (Q1). Breast cancer survivors are older than the average participant, consume fewer Kcal per day, a higher percentage of the sample are diabetic (31.4% vs. 14.8% full sample), and a lesser proportion are smokers.

**TABLE 1:** Demographic, anthropometric, metabolic parameters of glucose and insulin, and behavioral characteristics of all participants, non‐cancer participants, cancer survivors, and breast cancer survivors from NHANES 2011 to 12 and 2013 to 2014.

Characteristics	All participants	Non‐cancer survivors	All‐cancer survivors	Breast‐cancer survivors	
Adiposity					
Sagittal abdominal diameter	22.4	22.3	23.1	24.0	(cm)
Waist circumference	97.5	97.2	100.0	101.8	(cm)
Body mass index	29.4	29.4	29.7	30.5	(kg/m^2^)
Overweight: BMI > 25	69.2	69.0	71.3	79.7	(%)
Obese: BMI > 30	41.6	41.4	44.3	48.3	(%)
Physical activity					
Q4 (moderate‐to‐vigorous)	223	227	187	179	(m/day)
Q3	223	223	219	217	(m/day)
Q2	223	223	227	228	(m/day)
Q1 (sedentary)	223	221	242	243	(m/day)
Monitor worn	14.9	14.9	14.6	14.4	(h/day)
Ethnicity					
Non‐Hispanic white	41.6	38.9	67.0	55.1	(%)
Black	23.2	24.4	12.1	16.9	(%)
Asian	11.2	11.9	5.0	6.8	(%)
Hispanic	21.2	22.0	14.1	19.5	(%)
Others	2.7	2.8	1.8	1.7	(%)
Education					
High school or below	40.2	40.6	36.5	40.7	(%)
Above high school	59.8	59.4	63.5	59.3	(%)
Income					
Hi	27.9	27.7	30.5	35.6	(%)
Mid	32.3	32.0	34.8	39.0	(%)
Lo	32.8	33.2	28.5	20.3	(%)
Age	49.0	47.7	61.6	65.9	(years)
Energy intake	1835	1846	1730	1643	(Kcal)
Diabetes or borderline diabetes	14.8	13.8	24.2	31.4	(%)
Current smoker	17.0	17.0	17.4	9.3	(%)
Observations	4068	3671	397	118	
Metabolism parameters					
Glucose	5.8	5.8	6.2	6.3	(mmol/L)
Observations—glucose	1796	1633	163	49	
Insulin	81.0	80.7	84.2	84.0	(pmol/L)
Observations—insulin	818	749	69	20	

### 2.4 Metabolism Parameters of Glucose and Insulin Levels‐Laboratory Methodology

A plasma fasting glucose and insulin laboratory analysis was conducted for a limited subset of the survey participants. While a fasting glucose analysis was conducted for both considered cycles by NHANES, the insulin analysis was only conducted for the 2011 to 12 cycle. Participants had their blood collected at the MEC by trained health technicians following an approximate 9‐h fasting period. Blood samples were processed, stored, and sent to Fairview Medical Center Laboratory at the University of Minnesota for analysis. Fasting plasma glucose levels were determined using the hexokinase enzymatic reference method. Insulin levels were measured using the Elecsys 2010 insulin chemiluminescent immunoassay, employing a “sandwich” technique with two specific monoclonal antibodies for human insulin. Table 1 also presents the average glucose and insulin levels for the study participants. Of the original sample with 4068 participants, glucose levels are reported for 1796, and insulin levels for 818. Breast cancer patients present higher levels of both glucose and insulin measures than the sample average.

### 2.5 Analysis

The analysis was conducted by regressing a participant's adiposity, and glucose and insulin metabolism parameters on PA levels. The main dependent variables were the natural logarithms of SAD, WC, BMI, glucose, and insulin. Each dependent variable was regressed individually against the estimate of moderate‐vigorous PA and against the estimate of sedentary PA, resulting in six regression models for the three adiposity measures and four for the glucose and insulin metabolism parameters. Daily moderate‐vigorous PA and sedentary PA were, respectively, measured as the average percentage of time a participant spent within the top or 4th quartile (Q4) and the lowest or 1st quartile (Q1). All models were adjusted by demographic and behavioral controls included in Table 1; for example, ethnicity, education, income, age, energy intake, diabetes, and smoker or non‐smoker. Last, the estimation identified if each participant was a cancer survivor, and if—additionally—the participant was a breast cancer survivor. Optimal regression trees, a methodology adopted from the machine learning literature and recognized for their predictive accuracy [37], were employed to assess the robustness of the main results of the analysis.

## 3 Results

### 3.1 Adiposity

Increases to both extreme quartiles of PA (Q4 and Q1) exhibited significant associations with changes to the three adiposity measures of the participants. Table 2 presents the coefficient estimates from regressing a participant's adiposity on their levels of PA and on behavioral and demographic characteristics (*N* = 4068). Robust standard errors are presented in brackets.

**TABLE 2:** Correlation coefficient estimates from the linear regression analysis of either moderate‐to‐vigorous (Q4) or sedentary [1] physical activity, and other covariates, on sagittal abdominal diameter, waist circumference, and body mass index.

	Moderate‐to‐vigorous (Q4)			Sedentary (Q1)		
	SAD	WC	BMI	SAD	WC	BMI
Physical activity	−0.05***	−0.0425***	−0.0512***	0.0727***	0.0631***	0.0889***
	[0.0063]	[0.0055]	[0.0083]	[0.0084]	[0.0071]	[0.0106]
Physical activity × cancer	−0.0012	0.0002	−0.0024	−0.0009	0.0001	−0.0028
	[0.0039]	[0.0033]	[0.0049]	[0.0035]	[0.003]	[0.0045]
Physical activity × cancer × breast	0.0168***	0.0084*	0.0138*	0.0104*	0.0045	0.0095
	[0.0063]	[0.0052]	[0.0078]	[0.0057]	[0.0047]	[0.0071]
Energy intake (Kcal × 1000)	0.0168***	0.0128**	0.0094	0.0164***	0.0125**	0.0099
	[0.0063]	[0.0055]	[0.0079]	[0.0063]	[0.0055]	[0.0078]
Diabetes	0.1055***	0.0861***	0.1086***	0.107***	0.0873***	0.1086***
	[0.0079]	[0.0066]	[0.01]	[0.0079]	[0.0066]	[0.0099]
Age > 60 yo	0.0445***	0.0186***	−0.0211**	0.0557***	0.0279***	−0.0109
	[0.0079]	[0.0069]	[0.0099]	[0.0077]	[0.0067]	[0.0096]
60 yo ≥ age > 50 yo	0.0799***	0.0499***	0.0436***	0.0832***	0.0527***	0.047***
	[0.0083]	[0.0072]	[0.0105]	[0.0083]	[0.0072]	[0.0104]
50 yo ≥ age > 40 yo	0.0504***	0.0385***	0.0459***	0.0527***	0.0404***	0.0483***
	[0.0085]	[0.0072]	[0.0103]	[0.0085]	[0.0072]	[0.0103]
Education: HS or above	−0.0212***	−0.0143***	−0.017**	−0.0238***	−0.0167***	−0.021***
	[0.0063]	[0.0054]	[0.0079]	[0.0063]	[0.0054]	[0.0079]
Income: High	−0.0384***	−0.0272***	−0.0352***	−0.0394***	−0.0281***	−0.0365***
	[0.0065]	[0.0055]	[0.008]	[0.0065]	[0.0055]	[0.008]
Ethnicity: Black	0.0722***	0.0317***	0.0814***	0.0749***	0.034***	0.0842***
	[0.0073]	[0.0065]	[0.0095]	[0.0073]	[0.0065]	[0.0095]
Ethnicity: Asian	−0.1374***	−0.123***	−0.1628***	−0.142***	−0.127***	−0.1677***
	[0.0087]	[0.0071]	[0.0102]	[0.0088]	[0.0072]	[0.0102]
Ethnicity: Hispanic	0.0088	0.0034	0.0372***	0.0052	0.0004	0.0344***
	[0.0077]	[0.0064]	[0.0094]	[0.0077]	[0.0064]	[0.0094]
Ethnicity: others, except non‐Hisp White	−0.0008	−0.0136	−0.0003	−0.0005	−0.0134	0.0001
	[0.0188]	[0.016]	[0.0223]	[0.0191]	[0.0161]	[0.0225]
Constant	3.0835***	4.5895***	3.4278***	2.6986***	4.2577***	2.9825***
	[0.0502]	[0.0437]	[0.0629]	[0.0564]	[0.0485]	[0.0703]
*R* ^2^	0.2229	0.1786	0.1585	0.2227	0.1794	0.1630
*R* ^2^adj	0.2202	0.1757	0.1556	0.2200	0.1765	0.1601
LogLikelihood	1304	1942	417	1304	1944	428
Obs	4068	4068	4068	4068	4068	4068

*Note:* The dependent variables are the natural logarithm of sagittal abdominal diameter, waist circumference and body mass index. The robust standard errors are in brackets. *, **, and *** denote statistical significance at the 10%, 5%, and 1%, respectively. All variables are defined in Section 2.

A 1% increase in daily moderate‐vigorous PA (Q4) was associated, on average, with a 0.05% decrease in SAD, a 0.0425% decrease in WC, and a 0.0512% decrease in BMI. Conversely, a 1% increase in daily sedentary PA (Q1) was associated, on average, with a 0.0727% increase in SAD, a 0.0631% increase in WC, and a 0.0889% increase in BMI.

The subset of all cancer survivors included in the sample did not exhibit distinct associations between adiposity and PA than those exhibited by all participants. That is, for all cancer survivors, the associations of the three adiposity measures to changes in the extreme quartiles of PA were not significantly different from the associations observed on these measures for the full sample. Controlling the regression for whether the participant is a breast cancer survivor revealed a different scenario among these participants. Increases in daily moderate‐to‐vigorous PA (Q4) were positively and significantly associated with changes to the three adiposity measures for breast cancer survivors, but at a lesser average rate than what was observed for the full sample. The associated decrease in SAD for breast cancer survivors to a 1% increase in daily moderate‐vigorous PA (Q4) is 0.0168% less than for all women in the sample. Similar moderated responses were observed for WC (0.0084%) and for BMI (0.0138%).

The positive associations of adiposity measures and daily sedentary PA (Q1) were magnified for breast cancer survivors, but with statistical significance only for SAD. A 1% increase in daily sedentary PA (Q1) in breast cancer survivors was associated with an additional increase of 0.0104% in SAD over the increases observed to all women in the sample.

The coefficient estimates for the behavioral and demographic covariates on the estimation of SAD, WC, and BMI were consistent with those previously reported in the literature. Most notably, a diabetes diagnosis was associated, on average and holding all other covariates constant, with increases of 8.6% to 10.9% in adiposity measures among participants. SAD and WC were positively and significantly correlated with caloric intake. The three adiposity measures appeared positively correlated with age. High education and high income exhibited negative relationships with the three adiposity measures. On average, possessing Asian ethnicity significantly decreased the three adiposity measures, whereas possessing Black ethnicity increased the three measures.

To assess the robustness of the results, machine learning methods were implemented in the analysis. Table 3 presents the importances of the covariates when SAD was estimated using optimal regression trees.^4^ In estimating the value of SAD among participants, sedentary PA (Q1) and moderate‐to‐vigorous PA (Q4) respectively contributed 9.8% and 10% to the model's estimation accuracy of SAD. Noteworthy is the finding that daily PA proved to have a higher importance coefficient in predicting SAD than the participant's daily caloric intake. The importances of both types of PA increased for cancer and breast cancer survivors when predicting SAD, supporting the results reported in Table 2. Diabetes was the most important covariate for predicting SAD, followed by being of Asian or Black ethnicity.

**TABLE 3:** Importance coefficients of moderate‐to‐vigorous PA (Q4) and sedentary PA (Q1), and other covariates, from the estimation of optimal regression trees on sagittal abdominal diameter.

	SAD moderate‐to‐vigorous (Q4)	SAD sedentary (Q1)
Physical activity	10.0%	9.8%
Physical activity × cancer	1.6%	2.6%
physical activity × cancer × breast	1.6%	2.6%
Energy intake (Kcal × 1000)	1.7%	3.3%
Diabetes	29.2%	24.1%
Age > 60 yo	2.4%	4.8%
60 yo ≥ age > 50 yo	0	0.2%
50 yo ≥ age > 40 yo	0	0
Education: HS or above	0	0
Income: High	0	0
Ethnicity: Black	11.8%	15.1%
Ethnicity: Asian	42.0%	34.7%
Ethnicity: Hispanic	0	0
Ethnicity: Others, except non‐Hisp White	0	0
Constant	0	0
*R* ^2^	0.1731	0.1960
Max depth	4	6
Min samples leaf	2	4

### 3.2 Metabolism Parameters of Glucose and Insulin

Increases in the extreme quartiles of PA (Q4 and Q1) were significantly associated with changes in the two metabolic parameters, fasting glucose and insulin levels, of the participants. Table 4 presents the coefficient estimates obtained from regressing a participant's glucose and insulin measures on levels of PA.

**TABLE 4:** Correlation coefficient estimates from the linear regression analysis of either moderate‐to‐vigorous (Q4) or sedentary (Q1) physical activity and other covariates on glucose and insulin.

	Moderate‐to‐vigorous (Q4)		Sedentary (Q1)	
	Glucose	Insulin	Glucose	Insulin
Physical activity	−0.0499***	−0.1734***	0.0405***	0.244***
	[0.0104]	[0.0491]	[0.0125]	[0.0735]
Physical activity × cancer	0.0042	0.0092	0.006	0.0152
	[0.0051]	[0.0307]	[0.0053]	[0.0297]
Physical activity × cancer × breast	−0.0036	0.0057	−0.0029	−0.0143
	[0.0084]	[0.0523]	[0.0089]	[0.0506]
Energy intake (Kcal × 1000)	−0.0002	0.1027*	−0.0026	0.0938*
	[0.0104]	[0.0561]	[0.0105]	[0.0563]
Diabetes	0.2966***	0.2577***	0.3034***	0.2712***
	[0.0221]	[0.0704]	[0.0223]	[0.0697]
Age > 60 yo	0.0555***	−0.092	0.0665***	−0.0577
	[0.0118]	[0.0616]	[0.0111]	[0.0604]
60 yo ≥ age > 50 yo	0.0635***	−0.0146	0.066***	−0.0089
	[0.0113]	[0.0649]	[0.0113]	[0.0655]
50 yo ≥ age > 40 yo	0.0414***	−0.0526	0.0432***	−0.0446
	[0.0112]	[0.0723]	[0.0112]	[0.0722]
Education: HS or above	−0.024**	−0.1432***	−0.0237**	−0.1457***
	[0.0094]	[0.0525]	[0.0094]	[0.0521]
Income: High	−0.0198**	−0.0756	−0.0207**	−0.0748
	[0.0085]	[0.0549]	[0.0086]	[0.0557]
Ethnicity: Black	−0.0051	0.2255***	−0.0036	0.2365***
	[0.0115]	[0.0592]	[0.0116]	[0.0591]
Ethnicity: Asian	0.0034	−0.073	−0.0002	−0.0894
	[0.0123]	[0.0676]	[0.0124]	[0.0675]
Ethnicity: Hispanic	0.0255**	0.1552**	0.0185*	0.1477**
	[0.0113]	[0.0613]	[0.0112]	[0.0612]
Ethnicity: Others, except non‐Hisp White	−0.0289	−0.138	−0.0281	−0.1298
	[0.0215]	[0.1851]	[0.0213]	[0.1831]
Constant	1.8261***	3.9784***	1.5555***	2.7187***
	[0.0864]	[0.4402]	[0.0924]	[0.4946]
*R* ^2^	0.34238	0.09422	0.33573	0.09388
*R* ^2^adj	0.33721	0.07843	0.33051	0.07808
LogLikelihood	615	−788	606	−788
Obs	1796	818	1796	818

*Note:* The dependent variables are glucose and insulin. The robust standard errors are in brackets. *, **, and *** denote statistical significance at the 10%, 5%, and 1%, respectively. All variables are defined in Section 2.

A 1% increase in daily moderate‐vigorous PA (Q4) was associated, on average, with a 0.0499% decrease in glucose and a 0.1734% decrease in insulin, while a percentage increase in daily sedentary PA (Q1) was associated with a 0.0405% increase in glucose and a 0.2440% increase in insulin. As observed in Table 4, the two metabolic parameters, glucose and insulin measures, of all cancer survivors and of breast cancer survivors did not exhibit distinct associations with PA compared to those exhibited by all participants.

As for the analysis of adiposity measures, diabetes exhibited a strong correlation with glucose and insulin measures among participants. A diabetes diagnosis was associated with increases of 25.8%–30.3% in the metabolic parameters. Age is positively correlated with the glucose measure in participants. Possessing Hispanic ethnicity was associated with significant increases in both metabolic measures, and a Black ethnicity was significantly associated with the insulin measures.

## 4 Discussion

Population‐based studies confirm increasing obesity rates over time among US cancer survivors, with breast cancer survivors showing the highest burden [10, 11]. The representative samples of the US population collected by NHANES reflect this tendency (Table 1), stressing the importance of continuously monitoring adiposity in breast cancer survivors. Previous works have shown that increased physical activity (PA) contributes to reductions in the key non‐invasive adiposity markers: SAD, WC and BMI [38, 39]. Our study finds that the associations between PA and the adiposity metrics among breast cancer survivors are complex and significantly distinct from those of non‐cancer cancer survivors and of other cancer survivors. A negative correlation exists between moderate‐to‐vigorous PA and all adiposity metrics for all participants, and the magnitudes of these associations are lesser for breast cancer survivors. When analyzing the opposing side of the PA spectrum, it is observed that the correlation of daily sedentary behavior and SAD is greater for breast cancer survivors than for all cancer survivors and for the full sample of women.

The lesser correlation of SAD among breast cancer survivors with moderate‐to‐vigorous PA and its greater correlation with sedentary behavior represent unique challenges for this population. Greater weight and adipose tissue concentration are linked to increased risks of cancer recurrence and markers such as SAD and WC are instrumental in evaluating specific health risks, particularly in diagnosing metabolic syndrome [29, 34, 40]. BMI—while commonly used to assess overall adiposity—is not an accurate marker of metabolic health; for instance, individuals with a BMI over 30 kg/m^2^ can be metabolically healthy, whereas those with a BMI below 25 kg/m^2^ may not be [6, 35]. WC focuses on abdominal fat but does not specifically measure visceral fat. SAD is a strong and reliable anthropometric indicator of visceral adipose tissue, as demonstrated by its high correlation with visceral fat measured by imaging techniques such as computed tomography (CT), MRI scans, and energy x‐ray absorptiometry [40, 41, 42, 43]. However, it is important to note that SAD does not measure visceral fat exclusively; it can also be influenced by subcutaneous fat, though to a lesser extent than other measures like WC [42, 44]. Still, SAD continues to be a preferred non‐invasive proxy for estimating visceral fat, which is closely linked to metabolic and cardiovascular risks [41, 45]. Studies have stressed that measuring WC presents challenges across diverse populations, especially among individuals with larger abdominal girths. In contrast, SAD demonstrates greater reliability across varying body sizes, making it a preferred method for patient evaluation [36, 46].

Breast cancer survivors exhibit higher levels of SAD compared to both non‐cancer individuals and survivors of other cancers, and physical activity alone appears to have reduced efficacy in reducing SAD in this population. Nevertheless, maintaining physical activity remains crucial for the overall health of cancer survivors, particularly those with a history of breast cancer [22, 31, 47]. Elevated SAD is associated with increased insulin resistance, emphasizing its role as a metabolic risk marker [48]. Our analysis suggests that breast cancer survivors, while possessing the highest levels on all adiposity markers, exhibit the greatest correlation of SAD with increases in a daily sedentary lifestyle, posing pronounced health risks [46, 49, 50]. Metabolic effects of cancer‐related treatments may play a role in this observation. Tamoxifen and other endocrine therapies, such as aromatase inhibitors, have been associated with increased fat accumulation and adverse effects on metabolic regulation [51, 52]. Although some studies have reported an increased risk of clinically significant weight gain among users compared to non‐users, this association has not been consistently observed across all studies or with all types of endocrine therapy [20]. Preclinical studies indicate that tamoxifen may exacerbate visceral adiposity and metabolic dysfunction, particularly when administered in the context of pre‐existing obesity or high‐fat diets. For instance, tamoxifen‐treated obese mice have shown increased adipocyte hypertrophy, hepatic steatosis, and impaired glucose tolerance compared to lean counterparts [53]. These effects are thought to be mediated by tamoxifen's modulation of estrogen signaling in adipose and hepatic tissues under conditions of metabolic stress.

Diabetes and borderline diabetes are pronouncedly higher within breast cancer survivors than among all cancer survivors and the general population (Table 1). Recent works have also reported on this strong correlation between new‐onset diabetes and breast cancer [54]. Insulin resistance—a precursor to type 2 diabetes—occurs when insulin is no longer effective at reducing blood glucose levels, triggering compensatory hyperinsulinemia and ultimately leading to the development of type 2 diabetes [15]. Dysregulation of insulin and glucose metabolism is a hallmark of metabolic dysfunction [55]. Studies have shown a correlation between SAD and dysglycemia [56], and breast cancer recurrence is more frequent in patients with higher levels of metabolic syndrome [48, 57, 58]. Furthermore, previous research indicates an elevated risk of type 2 diabetes among breast cancer survivors, particularly in those treated with tamoxifen [18].

PA plays a crucial role in regulating insulin and blood sugar levels, leading to improvements in glycemic control, reduced variability in blood sugar levels, and decreased insulin resistance. Our analysis confirms the benefits associated with PA in managing insulin and glucose levels across all participants, including breast cancer survivors. We uncover an inverse relationship between insulin and glucose levels and moderate‐to‐vigorous PA. This relationship is particularly important as insulin activates multiple pathways involved in aggressive breast cancer biology, including PI3K (Phosphoinositide 3‐kinase)/Akt (Protein kinase B) and (Mitogen‐activated protein kinase) pathways, which regulate cell growth, proliferation, survival, and metabolism [59, 60]. Hyperinsulinemia and insulin resistance are associated with reduced glucose uptake, increased fatigue, and aggravated insulin resistance in muscle tissue. Moreover, in adipose tissue, hyperinsulinemia stimulates lipid accumulation [6, 61, 62, 63].

Advising breast cancer patients on weight management and lifestyle changes presents significant challenges for clinicians, unlike the structured protocols of chemotherapy. The absence of a standardized algorithm for personalized lifestyle interventions further complicates this task. However, structured programs that incorporate exercise, diet, and counseling show promise in significantly improving post‐treatment outcomes [9]. Our findings highlight the intricate and complex relationship between physical activity, sedentary behavior, adiposity, metabolic health, and breast cancer survival. It is essential to implement comprehensive lifestyle modifications that prioritize PA alongside other strategies. These modifications should include dietary interventions and targeted metabolic health strategies to monitor and reduce SAD, as well as regulate insulin and glucose metabolism to prevent metabolic dysfunction. By integrating daily moderate‐to‐vigorous PA with these approaches and limiting a sedentary lifestyle, patients may achieve more holistic and effective improvements in overall health outcomes. Since PA is a modifiable lifestyle risk factor, it represents an immediate and evidence‐based intervention that could benefit many breast cancer patients, and it can be readily implemented.

This research analyzes the most recent objectively measured PA assessment of a nationally representative sample, and quantifies the associations between physical activity levels, adiposity measures, and metabolic health parameters. There are known limitations to this study. One limitation of our approach is that participants self‐reported their breast cancer status without specifying disease stage or treatment regimen. Future research should carefully address this limitation to enhance our understanding in this field. Additionally, the study relies on making inferences about the actual PA of each participant based on the nine‐day observational period employed by NHANES. Extensions to the study would benefit from a longer duration period and multiple samplings throughout the year, and a more detailed classification of the nature of the actual moderate‐to‐vigorous and sedentary PA performed by the participants. Lastly, a further extension of this work would involve randomized controlled trial experiments where changes in PA are objectively measured using accelerometers and their impact on adiposity and metabolic parameters is carefully recorded.

## 5 Conclusion

In summary, this study provides an updated assessment of physical activity among breast cancer survivors using the most recent objectively measured PA assessment of the NHANES nationally representative sample. The results confirm rising obesity rates among US breast cancer survivors, highlighting the need for ongoing adiposity monitoring. Daily moderate‐to‐vigorous physical activity effectively reduces glucose and insulin levels, which are crucial for metabolic health. It is vital to implement comprehensive lifestyle modifications that emphasize physical activity, dietary changes, and targeted interventions to manage SAD. By combining these strategies and minimizing sedentary behavior, patients can enhance their overall health outcomes, which has significant public health implications.

## Author Contributions

C.d.l.P. is responsible for the conception and design of the study. J.J.C.‐A. extracted, managed, and analyzed the data. C.d.l.P. and J.J.C.‐A. wrote the original draft of the manuscript and revised it based on the recommendations contained in the Referee Report. E.F.L. and M.S. contributed to researching the literature, reviewing, and editing. All authors read and approved the final manuscript.

## Ethics Statement

The survey protocol was approved by the Ethics Review Board of the National Center for Health Statistics.

## Consent

All participants have signed a written informed consent form. https://wwwn.cdc.gov/nchs/nhanes/continuousnhanes/documents.aspx?Cycle=2011‐2012. https://wwwn.cdc.gov/nchs/nhanes/ContinuousNhanes/Documents.aspx?BeginYear=2013.

## Conflicts of Interest

The authors declare no conflicts of interest.

## Data Availability

The data that support the findings of this study are available on request from the corresponding author. The data are not publicly available due to privacy or ethical restrictions.

## Endnotes

^1^
The authors adopted the terminology promoted by the American Cancer Society where a *cancer survivor* describes “anyone who has a history of cancer, meaning they've had a cancer diagnosis and could be receiving treatment or have completed it.” *Source:* https://www.cancer.org/research/cancer‐facts‐statistics/survivor‐facts‐figures.html.

^2^

*Metabolic syndrome* is diagnosed when an individual meets at least three of the following criteria: waist circumference ≥ 80 cm, triglycerides ≥ 150 mg/dL, HDL cholesterol < 40 mg/dL, blood pressure ≥ 130/85 mmHg, or fasting glucose ≥ 100 mg/dL.

^3^
NHANES dataset is accessible at: https://wwwn.cdc.gov/nchs/nhanes/.

^4^
The authors thank an anonymous referee for suggesting the inclusion of this analysis to the manuscript.
