# Correction to “Objective Assessment of Physical Activity in Breast Cancer Survivors: Associations With Adiposity and Metabolic Parameters of Glucose and Insulin—Insights From NHANES”

**DOI:** 10.1002/cnr2.70424

**Published:** 2025-12-09

J. J. Cao‐Alvira, E. F. Luciano, M. Sauane, and C. de la Parra, “Objective Assessment of Physical Activity in Breast Cancer Survivors: Associations With Adiposity and Metabolic Parameters of Glucose and Insulin—Insights From NHANES,” *Cancer Reports* 8, no. 9 (2025): e70339, https://doi.org/10.1002/cnr2.70339.

In the published article, affiliation 5 is incomplete. It currently reads:

5 City University of New York, New York, New York, USA

It should read:

5 Ph.D. Programs in Biochemistry and Biology, The Graduate Center, City University of New York, New York, New York, USA

We apologize for this error.

